# Digital Health Interventions in Children and Adolescents With Type 1 Diabetes Mellitus and Their Impact on Clinical and Behavioral Outcomes: Scoping Review

**DOI:** 10.2196/79338

**Published:** 2026-03-02

**Authors:** Eirena Beh, Jiaying Lin, Geraldyn Leong, Pei Yi Loh, Ee Jin Poh, Daniel Chan

**Affiliations:** 1 Department of Paediatric Medicine, KK Women's and Children's Hospital, 100 Bukit Timah Road, Singapore, 229899, Singapore, 65 87900567; 2 Paediatrics Academic Clinical Programme, KK Women's and Children's Hospital, Singapore, Singapore; 3 Lee Kong Chian School of Medicine, Nanyang Technological University, Singapore, Singapore; 4 Endocrinology Service, KK Women's and Children's Hospital, Singapore, Singapore; 5 Duke-NUS Medical School, Singapore, Singapore

**Keywords:** mhealth, mobile health, digital health, eHealth, pediatrics, diabetes, diabetes mellitus, type 1 diabetes, pediatric diabetes

## Abstract

**Background:**

Effective self-management of type 1 diabetes mellitus (T1DM) by children and adolescents remains challenging despite advances in insulin delivery and glucose monitoring technologies. Mobile health (mHealth) interventions have emerged as promising tools to support pediatric diabetes care. However, their clinical impact and the behavioral mechanisms through which they operate—particularly those grounded in social cognitive theory (SCT)—are not well established.

**Objective:**

This scoping review assesses mHealth apps focused on the management of T1DM in the pediatric population and looks into the underlying behavioral frameworks in accordance with SCT.

**Methods:**

We conducted a scoping review of 5 databases (PubMed, Cochrane Library, EMBASE, CINAHL, and Scopus) for English-language studies published between January 2000 and July 2024. Eligible studies evaluated mHealth apps for children and adolescents with T1DM (≤18 years) and reported outcomes including glycemic control, self-efficacy, adherence, self-management, or quality of life. Data were extracted and synthesized according to clinical outcomes and the presence of SCT constructs, namely self-efficacy, behavioral capability, expectations, reinforcements, and reciprocal determinism.

**Results:**

Of 5607 studies screened, 12 met the inclusion criteria. These comprised 4 randomized controlled trials, 4 pilot studies, 2 pre-post intervention studies, 1 retrospective cohort study, and 1 double crossover trial. App features included glucose logging, insulin tracking, bolus calculators, reminders, gamification, and structured educational content. Hemoglobin A_1c_ (HbA_1c_) outcomes were reported by 9 studies; 4 demonstrated statistically significant improvements, while the others reported stability or no change. Several studies also reported improvements in treatment adherence and perceived self-efficacy. Of the 12 studies, 11 incorporated at least 1 SCT construct, with most integrating behavioral capability and self-efficacy as core components. Interventions using multiple SCT constructs showed greater promise for supporting sustained behavior change.

**Conclusions:**

mHealth apps for pediatric T1DM are complex behavioral interventions that often leverage key principles of SCT to promote effective self-management. Although the evidence supports modest benefits in glycemic control and behavioral outcomes, heterogeneity in study design and outcome measurement limits broader generalizability. Future research should prioritize the development and evaluation of SCT-informed digital interventions with standardized outcome frameworks to improve pediatric diabetes care.

## Introduction

Type 1 diabetes mellitus (T1DM) is a chronic autoimmune condition characterized by the destruction of insulin-producing pancreatic β-cells, leading to insulin deficiency and hyperglycemia. T1DM is among the 5 most common chronic illnesses in children attending school [1]. Findings observed from the SEARCH for Diabetes in Youth study [2] noted the prevalence of T1DM in youth aged 0 years to 19 years increased by 21.1% between 2001 and 2009, with an annual incidence growth rate of 1.4% reported between 2002 and 2012. This increase was noted across various demographic and ethnic groups.

T1DM management carries a substantial burden—including clinical, cognitive, and psychosocial—for both children and their caregivers, requiring daily insulin administration; frequent blood glucose monitoring; precise dietary management; and ongoing adherence to a structured, health-oriented lifestyle. Advancements in technologies in the treatment of T1DM such as insulin pumps, continuous glucose monitoring (CGM) systems, and intermittently scanned CGM systems have significantly improved patients’ metabolic control and, in turn, have helped to mitigate long-term diabetic complications [3].

Despite advances in insulin delivery systems and glucose monitoring technologies, achieving optimal glycemic control continues to rely heavily on effective self-management. Effective management requires strict and sustained discipline in regular glucose monitoring, carbohydrate counting, and adherence to an insulin regimen. This remains a long-term challenge, particularly among children and adolescents as they navigate emerging autonomy in the self-management of their diabetes [4]. With the advent of digital health, patients and health care providers can now leverage digital tools such as video conferencing, mobile health (mHealth) apps, and remote monitoring systems to improve accessibility to consistent health care, revolutionizing the management of T1DM. Recognizing its potential, the American Diabetes Association advocates that telehealth should complement in-person visits to achieve optimal glycemic management [5]. As such, exploring effective and innovative strategies that enhance self-management skills in the pediatric population with T1DM becomes critical. With the rapid advancement of mHealth technologies, mHealth apps have emerged as promising tools to support disease management by children and adolescents with T1DM. These digital interventions integrate features such as real-time reminders, interactive education, gamification, and data sharing to promote treatment adherence and self-management. Although several studies suggest that mHealth apps may improve glycemic control and psychosocial outcomes in pediatric T1DM, the extent and consistency of their clinical and behavioral impact remain unclear.

Social cognitive theory (SCT) [6,7] is a widely applied behavioral framework that explains how individuals acquire and maintain health behaviors through the dynamic interaction of personal factors, behavioral patterns, and environmental influences—a concept known as reciprocal determinism. Key constructs include self-efficacy, behavioral capability, expectations, and reinforcement, all of which are critical to sustaining self-management in chronic conditions such as T1DM. This scoping review aimed to synthesize the current evidence on mHealth interventions for pediatric T1DM, with a focus on their effects on key outcomes including hemoglobin A_1c_ (HbA_1c_), self-efficacy, treatment adherence, and quality of life. In doing so, we also examined the extent to which SCT constructs are embedded within these interventions to better understand how digital tools are designed to promote behavior change and support sustainable improvements in pediatric diabetes care.

## Methods

We conducted our scoping review with reference to the updated Joanna Briggs Institute guidance for scoping reviews. This was also aligned with the PRISMA-ScR (Preferred Reporting Items for Systematic Reviews and Meta-Analyses Extension for Scoping Reviews) guidelines for reporting scoping reviews [8]. The review protocol was preregistered on the Open Science Framework.

### Data Sources and Search Strategy

We conducted a comprehensive literature search in 5 databases including PubMed, Cochrane Library, EMBASE, CINAHL, and Scopus. A broad search strategy was used and included the following terms as MeSH, EMBASE Subject Headings terms, major headings, or title or abstract keywords: (“diabetes mellitus”) AND (“pediatrics” OR “child” OR “adolescent” OR “infant” OR “minor” OR “puberty” OR “schools”)) AND (“mobile applications” OR “telemedicine” OR “telehealth” OR “mobile health”).

Search results were limited to studies published in English between January 2000 and July 2024. This time frame ensures the inclusion of studies reflecting contemporary technological capabilities and behavioral frameworks relevant to mHealth interventions. The studies were screened and selected by 3 independent reviewers (EB, JL, and GL) and adjudicated by an independent fourth reviewer (DC).

### Screening and Data Extraction

We used the web-based software Covidence [9] for title and abstract screening, full-text review, and subsequent data extraction. We extracted the following information about each study: author, year of publication, study design, sample size, type of mHealth intervention, and main outcome findings.

### Eligibility Criteria

We included specific study designs such as randomized controlled trials, observational studies, and prospective interventional studies published between January 2000 and July 2024. We focused on studies reporting on the impact of mHealth apps on children and adolescents with T1DM. The definition for a pediatric population in this review was ≤18 years old.

We looked at important outcomes related to T1DM including HbA_1c_, self-efficacy, adherence to therapy, T1DM self-management skills, and quality of life. T1DM self-management included carbohydrate counting and insulin dosing, treatment of hyperglycemia, and prevention and treatment of hypoglycemia. Quality of life measurements were assessed objectively using validated questionnaires, including items assessing satisfaction with lifestyle, impact on life and relationships, and future concerns.

We excluded feasibility trials, study protocols, design studies, commentaries, case reports, and studies that were not published in English.

### Data Synthesis and Analysis

We conducted a thematic analysis using a deductive, semi-iterative approach guided by predefined constructs from SCT. Interventions were categorized and mapped to the 5 core SCT constructs: reciprocal determinism, behavioral capability, reinforcement, expectations, and self-efficacy. This analysis was performed by our team of reviewers independently (EB, JL, GL, PYL, and EJP), with disagreements first discussed and resolved among reviewers followed by further referral for adjudication by DC. We also assessed the reported impact of each intervention on the clinical and behavioral management of pediatric T1DM.

## Results

### Overview

**Figure 1.**
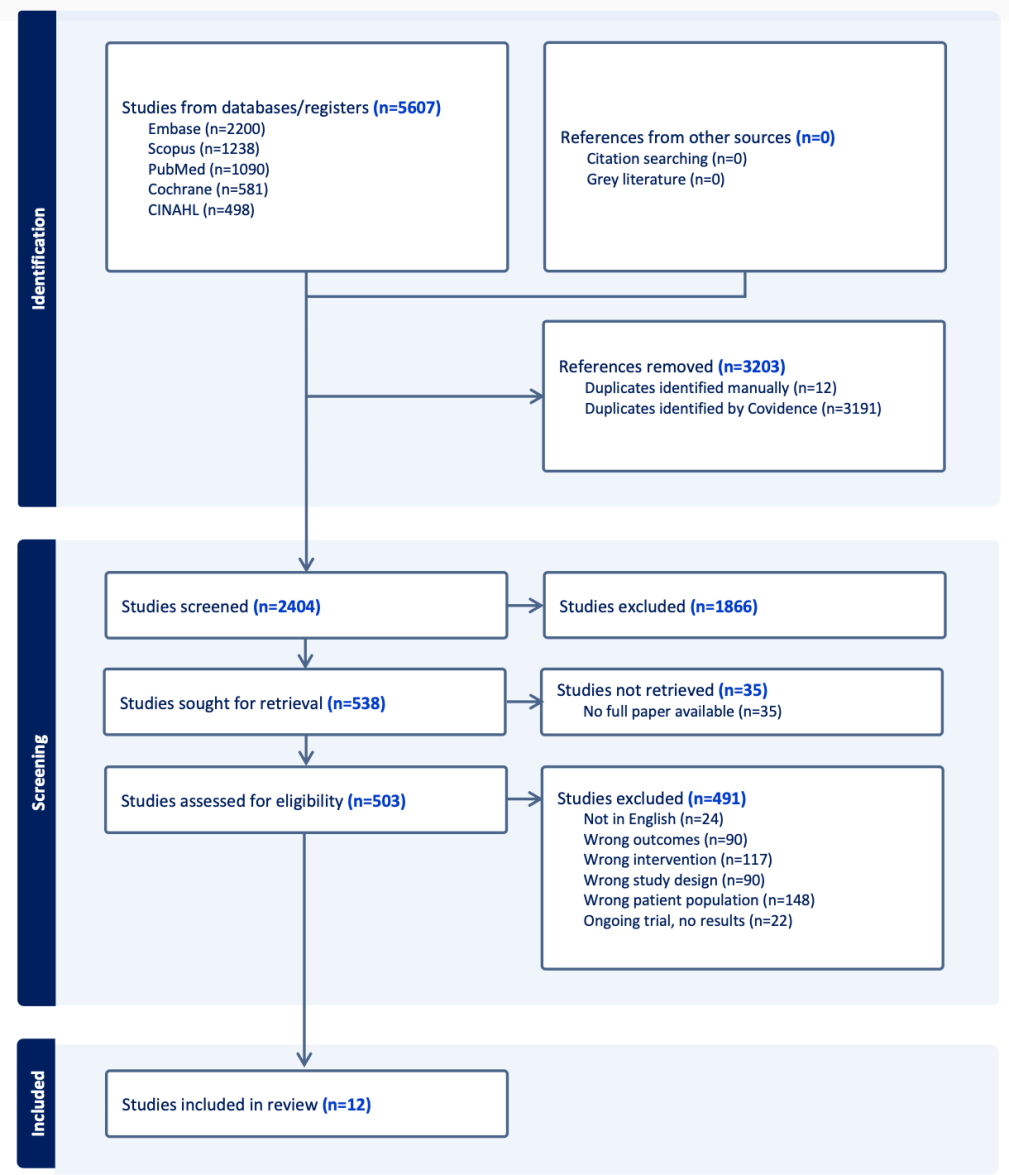
PRISMA (Preferred Reporting Items for Systematic Reviews and Meta-Analyses) flow diagram of study selection for the scoping review on mobile health (mHealth) interventions for pediatric type 1 diabetes mellitus.

Our database search, conducted in July 2024, yielded a total of 5607 studies. After removal of duplicates, there were 2404 studies. Through screening of title and abstracts, 1866 papers were excluded. A further 491 studies were excluded after full-text review. The screening and selection process is illustrated in the PRISMA flowchart (Figure 1).

We identified a total of 12 suitable studies. Of the 12 papers, 4 were randomized controlled trials, 4 were pilot studies, 2 were pre-post intervention studies, 1 was a retrospective cohort study, and 1 was a randomized double crossover study. Table 1 gives an overview of the included studies. Different diabetes mHealth apps were evaluated across the studies. App features included glucose level recording, insulin dose recording, feedback on glucose measurements, physical activity promotion, daily reminders, educational features, and reward systems. Some apps also combined multiple features on the platform.

**Table 1.** Study characteristics.

Domain	Author (year)	Title	Study design	Participants, n	Age (years), range	Outcome measures	App features
B^a^, R^b^, E^c^, S^d^	Cafazzo et al (2012) [10]	Design of an mHealth^e^ app for the self-management of adolescent type 1 diabetes: a pilot study	Pilot study	20	12‐16	Frequency of daily blood glucose readings, HbA_1c_^f^, self-care inventory survey, and Diabetes Family Responsibility Questionnaire	Gamification incentives and blood glucose trend analysis
B, E, S	Nilsson (2016) [11]	How young people can learn about newly diagnosed type 1 diabetes	Prospective pilot study	15	6.5‐16	Qualitative interview regarding knowledge and learning	App with educational content about injection technique, hyperglycemia, and hypoglycemia; diabetes game; an anatomy app; and a carbohydrate counter provided during the hospital stay
B, R, S	Goyal et al (2017) [12]	A mobile app for the self-management of type 1 diabetes among adolescents: a randomized controlled trial	RCT^g^	92	11‐16	HbA_1c_, hypoglycemic event, self-monitoring of blood glucose, and self-initiated adjustments to regimen	Out-of-range blood glucose trend alerts, coaching around out-of-range trend causes, and fixed point-based incentive system
B, S	Clifton et al (2018) [13]	A tablet-based educational tool: toward more comprehensive pediatric patient education	Prospective pilot study	106	6‐17	Perceived diabetes knowledge	Education regarding insulin regime, doses, treatment of hyperglycemia, prevention and treatment of hypoglycemia, and to teach patients and caregivers
B, R, E, S	Klee et al (2018) [14]	An intervention by a patient-designed do-it-yourself mobile device app reduces HbA_1c_ in children and adolescents with type 1 diabetes: a randomized double-crossover Study	Randomized double crossover	55	10‐18	HbA_1c_, QoL^h^, and hypoglycemia	Logging blood glucose readings, carbohydrate/sugar intake, and insulin dosing and regular, remote feedback from the diabetes care team
B, R, E, S	Pramanik et al (2019) [15]	Smartphone app as motivational intervention to improve glycemic control in adolescents with type 1 diabetes	Pre-post intervention study (single group)	34	11‐18	HbA_1c_	Daily reminders for insulin, meals, and exercise; thrice-daily reminders (morning, evening, bedtime) on weekdays; and an additional midday reminder on weekends for insulin administration, meal timing, and exercise
R	Hilliard et al (2020) [16]	Type 1 Doing Well: pilot feasibility and acceptability study of a strengths-based mHealth app for parents of adolescents with type 1 diabetes	RCT	80	12‐17	HbA_1c_ (%) and qualitative interviews	Praise messages and tracking diabetes-related behaviors with a weekly summary report
B, S	Alfonsi et al (2020) [17]	Carbohydrate counting app using image recognition for youth with type 1 diabetes: pilot randomized control trial	RCT	46	8.5‐17	HbA_1c_, carbohydrate counting accuracy, counting errors, QoL for youth, self-care, and patient or parent responsibility	Food identification and carbohydrate counting
B, R, E, S	Holtz et al (2021) [18]	An mHealth-based intervention for adolescents with type 1 diabetes and their parents: pilot feasibility and efficacy single-arm study	Prospective pilot study	30 (adolescent-parent pairs)	10‐15	Diabetes care adherence, QoL, family conflict, and HbA_1c_	Parent-adolescent communication
B, R, S	Kime et al (2023) [19]	Evaluation of the DigiBete App, a self-management app for type 1 diabetes: experiences of young people, families, and healthcare professionals	Pre-post intervention study (single group)	1343	5‐16	Qualitative interviews	Educational advice and resources such as guidance, quizzes, and educational and instructional videos; store insulin ratios/doses; and communication with the diabetes team via the app
B, S	Bender et al (2024) [20]	Impact of Sten-O Starter on glycemic management in children and adolescents with type 1 diabetes in the north region of Denmark	Retrospective cohort study	181	≤18^i^	HbA_1c_, time in range, time above range, and time below range	Educational app (each topic with animation, quizzes, and games) and instruction videos
B, R, E, S	Holtz et al (2024) [21]	The effect of an mHealth intervention for adolescents with type 1 diabetes and their parents	RCT	33 (adolescent-parent pairs)	10‐15	HbA_1c_, self-management, Diabetes Behavior Rating scale, and QoL via Impact on Family Scale	Parent-child communication

a B: behavioral.

b R: reinforcement.

c E: expectations.

d S: self-efficacy.

e mHealth: mobile health.

f HbA_1c_: hemoglobin A_1c_.

g RCT: randomized controlled trial.

h QoL quality of life.

i Inclusion criterion for the study.

The results for each of the included outcomes are described in the following sections.

### HbA_1c_

HbA_1c_ values as an outcome measure were reported in 9 studies [10,12,14-18,20,21]. In these studies, there were 355 people in the intervention groups and 208 people in the control groups. Significant improvement in HbA_1c_ values (reduced HbA_1c_ values) were reported within the intervention groups in 4 studies (Alfonsi et al [17]: −0.35%, *P*=.03; Klee et al [14]: –0.33%, *P*=.048; Pramanik et al [15]: −0.91%, *P*=.004; Goyal et al [12] −0.58%, *P*=.02). Significant differences were not found in 3 studies [10,16,20]. HbA_1c_ stability in the intervention group (HbA_1c_: −0.22%, *P*=.26) compared with worsening in the control group (HbA_1c_:+0.52%, *P*=.02) was reported in 1 study [21].

Holtz et al [18] did not find a significant change in HbA_1c_ levels between the groups; however, a higher use of the mobile app correlated with a greater improvement in HbA_1c_ levels (*P*<.005).

### Hypoglycemia

The occurrence of hypoglycemic events as an outcome measure was reported in 3 studies [12,20,21].

Bender et al [20] found a significant difference in the reduction of the time below range between the intervention and control groups at both 6 months (1.2% vs 2.6%, *P*=.02) and 12 months (1% vs 2%, *P*=.05) posttreatment. Klee et al [14] and Goyal et al [12] did not find any significant differences in hypoglycemic events.

### Self-Efficacy

Self-efficacy as an outcome measure was reported in 3 studies [13,16,17].

Alfonsi et al [17] reported increased carbohydrate counting accuracy (*P*=.008) and reduced frequency of individual counting errors >10 g (*P*=.047).

Hilliard et al [16] used the Diabetes Strengths and Resilience Measure for Adolescents to assess how often patients engaged in T1DM-related behaviors, but found no significant differences between the groups.

Through a multiple-choice survey designed with a 5-point Likert-type scale, Clifton et al [13] assessed the change in caregivers’ knowledge level for T1DM in general, hypoglycemia, hyperglycemia, and diabetic ketoacidosis. They found that 57% (36/63) of the intervention group perceived an increase in diabetes knowledge compared with 39% (16/41) of the control group, though the difference did not reach statistical significance (*P*=.059).

### Treatment Adherence

The impact on treatment adherence was evaluated in 4 studies [10,12,18,21].

Holtz et al [18] and Hilliard et al [16] used the Diabetes Behavior Rating Scale, showing significant improvements in diabetes behavior 12 weeks postintervention (*P*=.02 in both studies).

Goyal et al [12] used the Self Care Inventory questionnaire to measure adherence to treatment recommendations but did not find any significant changes in scores. Similarly, Cafazzo et al [10] did not find any significant changes in Self Care Inventory scores but did report a 50% (2.4 to 3.6 per day) increase in the daily average frequency of blood glucose measurements (*P=*.006).

### Quality of Life

Quality of life was measured through various questionnaires in 7 studies [10,12,16-19,21]. Alfonsi et al [17], Cafazzo et al [10], and Goyal et al [12] used the Diabetes Quality of Life for Youth questionnaire. Hilliard et al [16] used the Monitoring Individual Needs in Diabetes Youth Questionnaire. Klee et al [14] used the Quality of Life for Youth questionnaire, and Holtz et al [21] used the PedsQl questionnaire. None of the studies found significant differences between the intervention and control groups.

Holtz et al [18] reported significant improvement in quality of life measured via the PedsQoL generic scale (before study: mean 4.02, SD 0.84; after study: mean 4.27, SD 0.73; *t*_24_=2.48, *P*=.01, *d*=0.32).

### Family Quality of Life and Family Conflict

The impact on quality of life in the family was evaluated in 5 studies [10,12,16,18,21].

Holtz et al [21] used the Impact on Family Scale in their study, showing that parents’ perceptions of quality of family life improved significantly for the intervention group (*P*=.02).

Hilliard et al [16] used the Diabetes Family Impact Scale, Holtz et al [18] used the Revised Diabetes Family Conflict scale, and Cafazzo et al [10] and Goyal et al [12] both used the Diabetes Family Responsibility Questionnaire to examine family conflict and parent-adolescent interaction around diabetes. However, no significant differences between intervention and control groups were found.

### SCT Constructs Across the mHealth Apps

**Figure 2.**
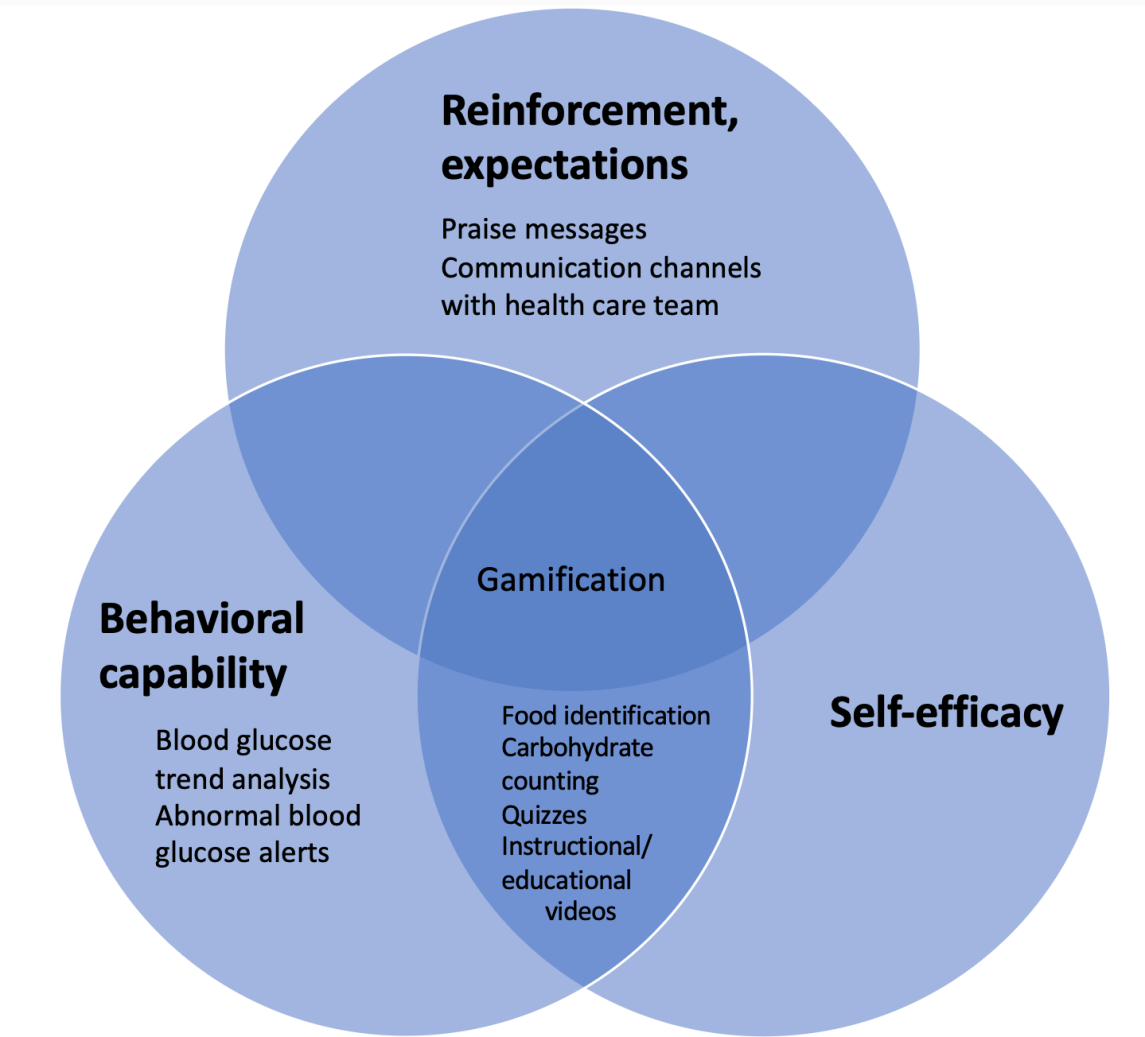
Mapping of mobile health (mHealth) app features to core constructs of social cognitive theory in pediatric type 1 diabetes management.

All included mHealth apps incorporated at least one SCT construct, with two-thirds of studies using at least 3 constructs within the same app. Among the individual SCT constructs, behavioral capability and self-efficacy were the most frequently used, appearing in 11 of the 12 included mHealth apps. Many of the apps had various tools: DigiBete [19] had the ability to store insulin ratios/doses and pump settings and guidance videos on the administration of injections, while WebDia [14] had a bolus calculator function with a section centered on carbohydrate estimation with pictorial assistance. In the iSpy study [17], the use of a mobile app designed to assist youth with T1DM with counting carbohydrates made food identification through images possible. Figure 2 illustrates the key features of the mHealth apps and their associated SCT construct(s).

Gamified features were used within the mHealth apps in 5 studies [10,11,18,20,21]. The Bant app [12] in particular was designed with a rewards algorithm that allocated game-like experience points for adherence to blood glucose testing with bonus points and level-ups awarded accordingly. A total of 161 rewards (an average of 8 rewards each) were distributed to participants based on their frequency of blood glucose measurement, with 50% (10/20) of participants collecting more than 10 awards. The use of gamification and rewards encouraged adherence and reinforced positive health behavior.

The majority of the studies provided educational modules to participants, thereby enhancing their behavioral capability. Alfonsi et al [17] and Klee et al [14] focused on nutrition with food identification and carbohydrate counting, while other studies [11-13,19] included analysis of blood glucose trends and awareness of hypoglycemia and hyperglycemia. Some studies also included educational materials regarding insulin storage, calculation, and administration. The DigiBete [19] and Sten-O Starter [20] apps also made use of quizzes to help users consolidate their learning and understanding of their own condition, with awards for completion that allowed for positive reinforcement.

### Intervention Engagement and User Analytics

Hilliard et al [16] reported parental use of the app for an average of 106.1 (SD 37.1) days over the 3-month study period, with parents logging in 1 or more times a day on 80% of the days. Alfonsi et al [17] reported 43% (9/21) of iSpy participants were still engaged (medium to high users) at the end of the study. Both noted high app acceptability ratings among their participants pertaining to usefulness, ease of use, and learning on the app.

Kime et al [19] reported an average of 4.4 videos viewed per app user. Uniquely, their app allowed for communication and interactions with health care professionals. It was noted that more than one-half of health care professionals surveyed (99/178, 55.6%) reported that they frequently or very frequently sent out information and news to support their patients and families via the clinic portal in the app.

Holtz et al [18] reported an average number of 63 (SD 27.7) days of app use, with 84% (21/25) of the participants having use in the medium or high use categories over the 90-day study period. Linear regression analysis demonstrated a significant effect between the overall use of the app and improvement in HbA_1c_ levels (*F*_1,20_=9.74, *P*<.005; *R*^2^=0.33).

Klee et al [14] shared that 80% (27/33) of participants reported using Webdia daily. However, the subgroup analysis showed that frequency of app use did not influence the effect of intervention, although overall results showed a significant difference in the decrease in HbA_1c_ between intervention and control groups.

Goyal et al [12] reported 35% (16/46) of the participants were moderately or highly engaged users (uploaded self-monitored blood glucose [SMBG] concentrations ≥3 days a week) over the 12-month period. Exploratory regression analysis demonstrated a statistically significant association between increased SMBG and improved HbA_1c_ in the intervention group. For a subgroup of users with SMBG≥5 times daily, there was a significant improvement in HbA_1c_ of 0.58% (*P*=.02) compared with the parallel control subgroup who did not have a significant HbA_1c_ change.

Holtz et al [21] reported an average of 23.3 (SD 16.44) days of parental app use and 57.72 (SD 25.4) days of adolescent app use over the 12-week study period. However, they did not identify any correlation between adolescents’ use days and HbA_1c_ levels.

Of the studies, 4 [11,13,15,20] did not report any usage data, as these were either interventions implemented within the health care setting or they were not included as a studied outcome.

## Discussion

### Principal Findings

We performed a scoping review to examine mHealth apps focused on the management of T1DM in the pediatric population, looking into their effects on clinical and patient-related outcomes, as well as examining the underlying behavioral frameworks informed by SCT. To our knowledge, no review has been conducted in the past decade to specifically examine pediatric digital health interventions for T1DM, with the most recent being published by Blake et al [22] in 2015. Although recent meta-analyses and umbrella reviews have evaluated mHealth interventions for diabetes [23,24], these reviews have primarily addressed adult populations or mixed-age cohorts, with limited focus on pediatric-specific outcomes. However, children and adolescents with T1DM face distinct developmental and psychosocial challenges that may influence both the design and effectiveness of digital interventions. Although Trnka et al [25] focused on categorizing coaching-based mHealth technologies for pediatric T1DM, our review builds on this by systematically evaluating a broader range of interventions, with specific attention to clinical outcomes such as HbA_1c_ and hypoglycemia, as well as behavioral outcomes including treatment adherence and self-efficacy. Furthermore, we uniquely analyzed the theoretical foundations of these interventions, drawing on constructs from SCT to elucidate the behavioral mechanisms underlying their impact.

**Figure 3.**
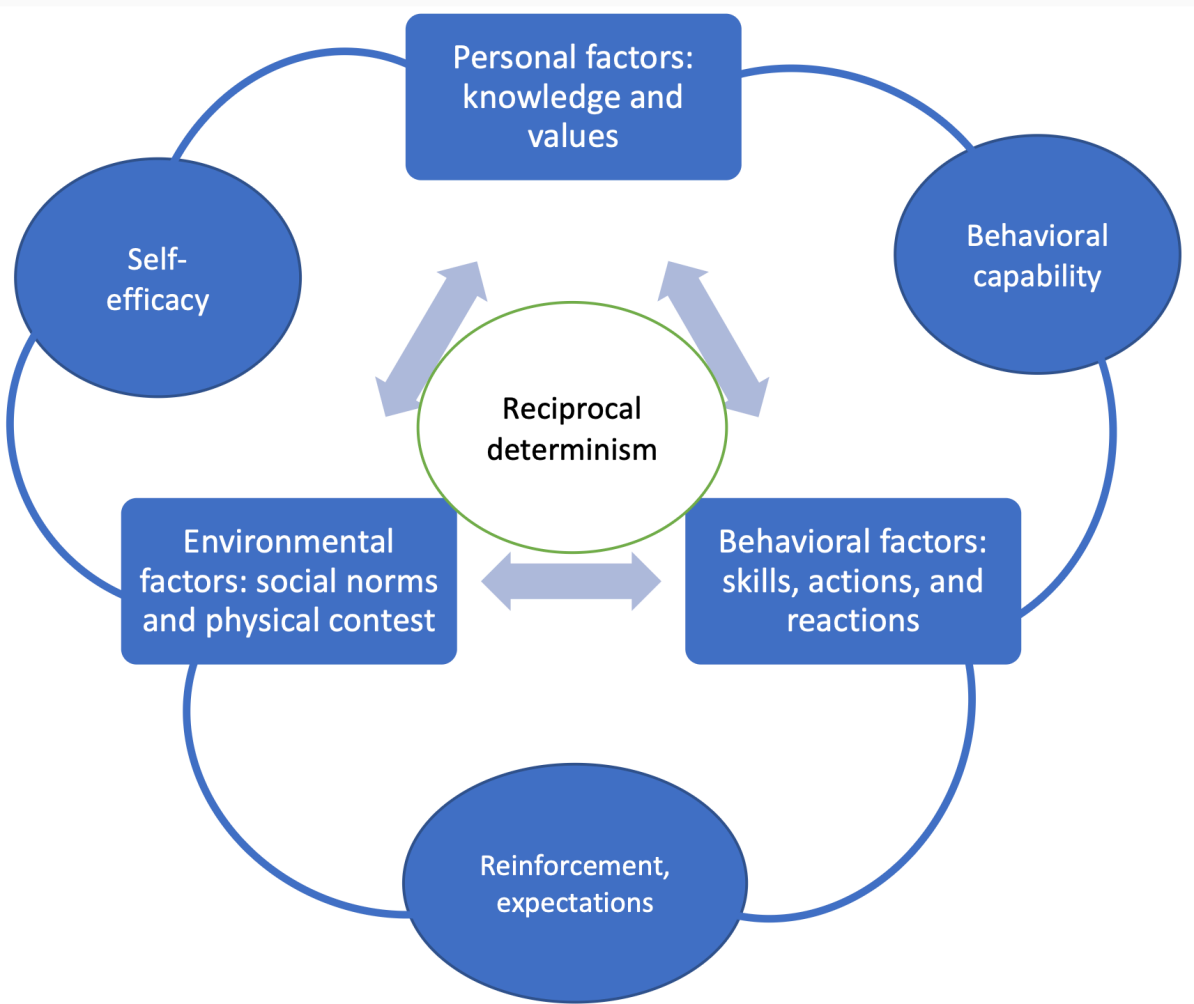
Triadic reciprocal causation and key elements of social cognitive theory.

The results of our study suggest that mHealth apps for children and adolescents with T1DM are complex interventions grounded in multiple constructs of SCT. A widely used framework in behavioral interventions [26], SCT [27] explains how behaviors are learned within a social context (Figure 3). This process is grounded in the interplay of personal factors, environmental influences, and behavior itself—a concept known as reciprocal determinism. Key constructs of SCT include (1) self-efficacy (belief in one’s ability to succeed), (2) behavioral capability (knowledge and skills to perform a behavior), (3) expectations (anticipated outcomes), and (4) reinforcements (responses that influence behavior recurrence) [6].

For reciprocal determinism, SCT proposes that behavior is influenced by 3 interlocking factors: personal, behavioral, and environmental factors [28]. Behavior is influenced by a continuous interaction between personal factors, environmental factors, and behavior itself.

Behavioral capability refers to an individual’s ability to perform a behavior, thus relying on their knowledge and skills [28]. The individual then learns from the consequences of their behavior.

Internal and external responses to a person’s behavior then positively or negatively reinforce the behavior. Expectations refer to the anticipated consequences one has of their behavior.

Self-efficacy refers to the level of a person’s confidence in their ability to successfully perform a behavior [29]. It is influenced by an individual’s own capabilities and environmental factors.

### Impact of mHealth on Clinical Outcomes in Pediatric T1DM

#### Glucose Control

mHealth apps for children and adolescents with T1DM could help improve glycemic control to varying degrees. Among the included studies, some mHealth apps were reported to significantly reduce HbA_1c_ over the course of exposure to the intervention, while others kept HbA_1c_ levels stable as opposed to control groups. This is consistent with observations in previous evidence, with umbrella reviews by Wang et al [24] and Kitsiou et al [23] showing pooled favorable effects of mHealth interventions on HbA_1c_, at least in the short term. This is a notable observation, as there are unique challenges for maintaining glycemic control in the pediatric population, ranging from hormonal fluctuations, conflicting academic commitments, and struggles with the growing responsibility of diabetes management to the impact of family and peer pressure [30]. Given the pervasiveness of smartphone use even among children and youths today [31], mHealth apps could potentially help by improving self-management ability and health literacy of children and adolescents, which tend to be poorer than that in adults [32], allowing for better treatment adherence and glycemic control. However, one should approach the matter with cautious optimism, as the effectiveness of mHealth apps remains heterogeneous across different studies and its sustained benefits in the long term remain unknown.

#### Educational Value and Treatment Adherence

mHealth apps can be of educational value by providing structured education that is easily accessible to patients and may facilitate improvement in communication between health care providers and patients, to align treatment steps and goals [33]. Many studies highlighted the usefulness of digital health interventions for improving patients’ and their families’ knowledge of their disease. From the DigiBete study [19], children and families with T1DM felt that the app was helpful for navigating the large volume of information available on T1DM and allowed them to retrieve information on T1DM readily. Of health care professionals, 83.7% (149/178) believed that the DigiBete app helped patients manage their T1DM better. Our findings corroborate with a previous cross-sectional study that found that educating children with T1DM and their parents regarding major complications of T1DM increased overall adherence to treatment and decreased their HbA_1c_ levels [34].

#### Family Interaction and Quality of Life

Studies have found that mHealth apps have the potential to improve family interactions as well as patients’ and family members’ perception of their quality of life. The MyT1DMHero app [18] linked adolescents with T1DM to their parents through two separate interfaces, allowing parents to establish a blood glucose testing schedule on the app so that the child could follow the schedule accordingly and update their blood glucose readings. Parents and children could send each other preprogrammed messages within the app to facilitate positive communication as well. The Type 1 Doing Well app [16], aimed at promoting supportive family diabetes management, allows parents to record positive T1DM-related actions that their child engaged in every day; ratings would be summarized and presented as a strengths summary for each family. There were multiple positive comments from families who felt that the app allowed them to generate a calmer atmosphere at home and helped adolescents feel that their efforts were recognized. Many of these apps, such as Webdia [14] and iSpy [17], received good feedback but did not show statistically significant differences in the quality of life from baseline and at postintervention. T1DM management can be particularly challenging for families, as it requires teamwork between parents and child [35] and conflicts can be common [36,37]. Ineffective communication can lead to youths feeling unsupported. mHealth technology offers a promising avenue to make behavioral interventions for family diabetes management more accessible [38,39]. The extent to which family dynamics and glycemic management are correlated is currently unknown, but the accumulated knowledge within a family has also been shown to have positive effects on glycemic management [40]. These studies show that, although mHealth apps are promising avenues to better improve family interactions and quality of life, more robust evidence is still required.

### SCT and its Application to mHealth

The majority of studies used at least 3 SCT constructs within the same app. This underscores the complex interplay between these elements, as they do not function in isolation but could reinforce one another in the design of mHealth apps and driving behavioral change. For instance, building behavioral capability such as equipping children with essential self-care skills could enhance self-efficacy [41], which in turn can strengthen expectations of improved health outcomes [42]. Similarly, reinforcements, such as gamified incentives, have been shown to sustain engagement while bolstering productivity and self-efficacy in pediatric T1DM management through tangible progress [43]. These combinations of constructs align with current taxonomies of the ideal app for people with diabetes [44], including tips and support, rewards, and reminders. Consequently, the dynamic interaction between these constructs also mirror reciprocal determinism, where personal confidence, environmental support (eg, caregiver involvement, app-based reminders), and self-management behaviors continuously influence one another [45].

Among individual SCT constructs, behavioral capability and self-efficacy were the most frequently used. Their prevalence reflects the emphasis of mHealth interventions on fostering self-management, which is in keeping with existing knowledge on the clinical efficacy of self-monitoring as a behavioral change technique in the management of diabetes [46]. This focus on self-management is particularly crucial in the management of diabetes in pediatric populations, where competency in self-care skills (from diet, exercise, and treatment adherence,to monitoring) from early in the life course could go a long way in supporting healthy growth and prevent the development of potentially debilitating complications [47,48]. mHealth interventions could help equip children with the necessary skills and reinforce long-term self-care behaviors while instilling confidence in their ability to manage their condition [49,50]. The spotlight cast on patient self-management also aligns with a broader shift toward patient empowerment, balancing autonomy with structured caregiver and health care provider support.

In this review, studies that used gamification and improved T1DM self-management demonstrated significant differences in clinical outcomes. The use of graphics, aesthetically colorful images, and avatars are important elements in mHealth apps that can make the apps more appealing to use and better engage children and adolescents. Gamification leverages SCT by incorporating its core constructs. For example, completing levels in a game and earning rewards build mastery, provide motivation, and boost self-efficacy. Gamification also works through positive reinforcement using points or social comparisons where users are rewarded for their performance and the relevance of their achievements are recognized on the gaming platform [51].

### Intervention Engagement

A key functionality of mHealth in clinical practice is improving clinical outcomes through behavior change and enhancement of patient adherence and treatment compliance. More frequent engagement is generally associated with improved treatment adherence or self-management [33].

Participant engagement and retention continue to be fundamental challenges for mHealth app studies [52]. Similarly, 2 of the included studies [17,18] reported initial high levels of usage followed by decreasing levels over time. This could be attributed to the “novelty effect” [53], whereby participants are more motivated to good performance when an experience is new to them. Additional feedback given for the mHealth apps included the lack of personalization [16], need for repeated manual entries [12,14], and requiring a secondary mobile or supplementary device [12].

Several studies [12,14,18] that reported data on user engagement noted medium to high usage levels; however, this did not necessarily translate to improved clinical outcomes. To examine the reasons for this gap, we considered research design and participant factors. Regarding the participant factors, the quantity of use might not reflect the quality of use. A user’s degree of engagement is influenced by the depth of the individual’s investment in the interaction with the digital tool, and this investment may be defined temporally, affectively, and/or cognitively [54]. Hence, current engagement measures are not able to fully detail user characteristics and their interactions with the app platform. For research design factors, there is a lack of standardized classification for user engagement. Studies tended to focus on a limited number of indicators of engagement, leading to obscuration of more complex patterns and posing a challenge for the generalizability of findings [55]. To address these factors, Shani et al [55] proposed an organizing framework to guide the measurement, reporting, and interpretation of engagement in digital intervention research. They suggested the conceptualization of digital interventions as a collection of stimuli (such as notifications and reminders) and tasks (such as opening the mobile app and practicing a relaxation technique) and considering user engagement with digital interventions as a continuous process. Moving forward, the adoption of such a standardized framework would be useful for measuring, reporting, and interpreting user engagement with digital interventions.

### mHealth in the Changing Landscape of T1DM Management

There have been transformative advances in technologies to support T1DM management including CGM devices, insulin pumps, and automated insulin delivery systems. There have been significant improvements in both glycemic control and quality of life measures for those who have integrated the use of these technologies into care of their diabetes [56].

Fang et al [57] reported a substantial increase in the use of CGM and insulin pumps among youths. However, the lowest rates of diabetes technology uptake and poorest glycemic control were observed among Hispanic, non-Hispanic Black, and state-insured youths and adults, and these disparities have persisted or even widened over time. Racial, ethnic, and socioeconomic disparities in access to diabetes technology have been identified as common barriers to uptake of diabetes technology in several studies [58-60]. This has also been compounded by individual-level barriers such as physical interferences from the device and body image concerns [61]. Studies [62,63] examining the psychosocial barriers to the uptake of diabetes technology have also highlighted varied user experiences with technical failures, fear of device dependence, and uncertainty of device accuracy. Diabetes care then defers back to SMBG and multiple daily insulin injections. Although CGM and insulin pump technologies are increasingly regarded as the standard of care in contemporary T1DM management, digital health interventions remain as both a valuable and accessible adjunct for individuals who either choose to delay the adoption of these technologies or simply lack access due to psychosocial or socioeconomic barriers. Such tools can still potentially support essential self-management behaviors including carbohydrate counting, insulin logging, and structured education. They could also play a complementary role in addressing educational, behavioral, and psychosocial needs that may not be the primary objective of these technologies.

Proprietary CGM manufacturers feature mobile apps that provide real-time glucose monitoring, trend alerts, and data sharing. Nonetheless, third-party apps such as mySugr and Glucose Buddy remain highly rated apps [64,65] on various mobile operating systems. Zivkovic et al [66] investigated the impact of transitioning from SMBG to CGM alongside the use of a mHealth app, mySugr, on users’ glycemic control. They found that this transition significantly improved glycemic control in terms of glucose variability and mean glucose levels. There remains an extensive selection of third-party apps catering to the various needs of those with T1DM, with features supporting data tracking, data analysis, food insights, and motivational support either in the form of online communities or chatbots. mHealth apps offer the potential of an integrated platform for seamless pairing with CGM sensors and devices while providing opportunity for real-time feedback, timely intervention, communication support (with family, health care providers, user community), and education about T1DM.

### Strengths and Limitations

This scoping review possesses several methodological and conceptual strengths. First, it represents the most up-to-date synthesis specifically focused on mHealth interventions for pediatric T1DM, addressing a critical evidence gap in the digital health literature. Unlike prior reviews that aggregated data across broader or mixed populations, this review targets children and adolescents—a group with unique behavioral, cognitive, and developmental needs that warrant tailored intervention strategies. The next notable strength lies in our use of deductive thematic analysis guided by an underpinning conceptual framework based on SCT. Intervention components were systematically mapped to key SCT constructs—self-efficacy, behavioral capability, reinforcement, expectations, and reciprocal determinism—allowing for an in-depth exploration of how behavioral change mechanisms are embedded in mHealth design. This theory-driven approach moves beyond descriptive synthesis to offer explanatory insight into how and why mHealth tools may support effective self-management in pediatric T1DM. Finally, by evaluating both clinical outcomes (such as HbA_1c_ and hypoglycemia) and behavioral outcomes (such as treatment adherence, self-efficacy, and quality of life), this review provides a comprehensive and translational understanding of the impact of mHealth interventions. The findings offer valuable guidance for the development and refinement of future digital health strategies that are both evidence-based and behaviorally grounded.

However, we do acknowledge that several limitations should be considered when interpreting the findings of this review. First, the overall number of eligible studies was limited, and many were studies with small sample sizes and short follow-up durations. This limits the statistical power and generalizability of the reported outcomes, particularly with respect to long-term clinical effectiveness. Next, due to the limited availability of high-quality randomized controlled trials, we included a range of nonrandomized study designs. Although this approach aligns with scoping review methodology and allows for broader coverage of real-world evidence, it introduces inherent risks of bias and limits the ability to draw causal inferences regarding intervention effectiveness. Third, there was substantial heterogeneity in outcome reporting across studies. Clinical and behavioral endpoints—such as HbA_1c_, adherence, and quality of life—were assessed using a variety of instruments, often with differing definitions, scales, or time points. This lack of standardization hinders direct comparisons across studies and precludes meta-analytic synthesis. Another notable limitation was the lack of inclusion of youths with T1DM as members of our research team to co-design the methodology of our work. Their lived experiences would also have provided deeper insights into contextualizing our findings and direct applicability of digital health. Using the key findings from this paper, our study team aims to involve children and young persons living with diabetes in subsequent qualitative and mixed methods studies examining the role of digital health interventions in both their lives as well as their families.

### Conclusion

In conclusion, mHealth apps for children and adolescents with T1DM are complex behavioral interventions that often incorporate multiple constructs from SCT. Current evidence suggests that these interventions may contribute to improved glycemic control, with several studies reporting stability or reductions in HbA_1c_ relative to control groups. Although the evidence base is heterogeneous, mHealth interventions show potential benefits across a range of behavioral and psychosocial outcomes, including self-efficacy, treatment adherence, quality of life, hypoglycemia management, and family dynamics. To strengthen the evidence and guide future implementation, further research is needed, using standardized outcome measures and robust methodological designs to better understand how theory-informed digital tools influence self-management and holistic well-being in pediatric T1DM.

## Supplementary material

10.2196/79338 Checklist 1 PRISMA (Preferred Reporting Items for Systematic Reviews and Meta-Analyses) checklist.

## Abbreviations

CGM: continuous glucose monitoring
HbA_1c_: hemoglobin A_1c_
mHealth: mobile health
PRISMA-ScR: Preferred Reporting Items for Systematic Reviews and Meta-Analyses Extension for Scoping Reviews
SCT: social cognitive theory
SMBG: self-monitored blood glucose
T1DM: type 1 diabetes mellitus
